# Advanced lung cancer inflammation index is associated with prognosis in skin cancer patients: a retrospective cohort study

**DOI:** 10.3389/fonc.2024.1365702

**Published:** 2024-10-11

**Authors:** Weifeng Lan, Wanli Zhuang, Ruiqi Wang, Xuewen Wang, Zhou Lin, Liqin Fu, Yanping Zhang, Yuqing Wen

**Affiliations:** ^1^ Department of Plastic Surgery, Longyan First Affiliated Hospital of Fujian Medical University, Longyan, Fujian, China; ^2^ Department of Gastroenterology, Jinjiang Municipal Hospital, Shanghai Sixth People’s Hospital Fujian, Quanzhou, Fujian, China; ^3^ Department of Gastroenterology, Xiamen Humanity Hospital of Fujian Medical University, Xiamen, Fujian, China; ^4^ Department of Histology and Embryology, School of Basic Medical Sciences, Fujian Medical University, Fuzhou, Fujian, China

**Keywords:** skin cancer, all-cause mortality, cancer mortality, advanced lung cancer inflammation index, NHANES

## Abstract

**Background:**

Skin cancer ranks as one of the most prevalent malignant tumors affecting humans. This study was designed to explore the correlation between the advanced lung cancer inflammation index (ALI), a metric that gauged both nutrition and inflammation statuses, in skin cancer patients and their subsequent prognosis.

**Methods:**

Data from the National Health and Nutrition Examination Survey (NHANES) spanning 1999-2018 were scrutinized, along with mortality tracking extending to December 31, 2019. Kaplan-Meier survival curves and COX regression analysis, utilizing NHANES-recommended weights, delineated the association between ALI levels and skin cancer prognosis. To decipher the potential non-linear relationship, a restricted cubic spline analysis was applied. Additionally, stratified analysis was conducted to affirm the robustness of our findings.

**Results:**

The 1,149 patients participating in NHANES 1999-2018 were enrolled. We observed a reverse J-shaped non-linear relationship between ALI and both skin cancer all-cause mortality and cancer mortality, with inflection points at 81.13 and 77.50, respectively.

**Conclusions:**

The ALI served as a comprehensive indicator of a patient’s nutrition and inflammation status and was demonstrably linked to the prognosis in skin cancer cases. The meticulous evaluation and continuous monitoring of these parameters in skin cancer patients bear clinical importance.

## Introduction

Skin cancer remains the most prevalent form of cancer, comprising approximately 8% of all cancers (1). Reports from the U.S. National Cancer Institute, utilizing data from the Surveillance, Epidemiology, and End Results (SEER) program, suggested that in 2022, an estimated 100,000 individuals in the U.S. were diagnosed with a form of skin cancer, with an anticipated 7,650 fatalities (2). There were three primary skin cancer types: basal cell carcinoma, squamous cell carcinoma, and melanoma. Basal cell and squamous cell carcinomas represented the majority of cases, whereas malignant melanomas were less common yet still significant in number. Malignant melanoma, known for its invasiveness, frequently metastasizes. Without early intervention, it could prove lethal. Despite advancements in skin cancer treatment over the last decade, outcomes for certain patients, particularly those with melanoma, remained suboptimal. To decrease mortality further, effective biomarkers were essential for clinicians to refine preventive and therapeutic strategies.

The concept of chronic inflammation as a critical component of the tumor microenvironment dated to 1828 and has been increasingly underscored by research (3, 4). The link between inflammation and cancer has become well-recognized. Studies indicated that an inflammatory milieu could accelerate tumor progression and foster an immune-suppressive environment. This was characterized by the recruitment of suppressive cells like CD4+, CD25+, FOXp3+ Treg (regulatory T cells) and included elements such as bone marrow-derived suppressor cells, tumor-associated macrophages, and regulatory dendritic cells. This recruitment was redundant and could be streamlined for clarity. Immunosuppression, mediated by factors like TGF-beta and IL-10, might facilitate immune evasion by tumor cells (5). Evidence consistently pointed to inflammation as a factor in skin cancer development (6, 7). Cancer-associated malnutrition, influenced by both the malignancy and its treatments (8), profoundly impacted patient prognoses. Notably, inflammation could diminish albumin levels and cause weight loss (9, 10), necessitating a more effective prognostic indicator that encapsulated the interplay between inflammation and nutrition. Cachexia in cancer patients was often the result of the chronic systemic inflammatory response and frequently indicated a poor outcome for cancer patients (11). Moreover, Sarcopenia, which has been reported to correlate with body mass index (BMI), was an important nutritional component of cancer cachexia syndrome (12).

The advanced lung cancer inflammation index (ALI) prognosticated outcomes across several cancer types, combining body weight, albumin, and neutrophil to lymphocyte ratio (NLR) to evaluate systemic inflammation (13). Studies corroborated ALI’s prognostic relevance in cancers like lung (14) and colorectal (15). ALI’s unique composition, which included both inflammatory and nutritional markers, could make it a superior systemic inflammation indicator. Yet, the scarcity of studies focusing on ALI’s relation to skin cancer prognosis remained.

This pioneering large-scale study investigated the ALI’s correlation with skin cancer, aiming to inform the condition’s diagnostic and therapeutic frameworks.

## Materials and methods

### Study population

National Health and Nutrition Examination Survey (NHANES) (16) was a nationally representative cross-sectional survey periodically conducted in the United States by the National Center for Health Statistics, employing a stratified multistage random sampling design. Our retrospective analysis utilized publicly accessible NHANES data spanning from 1999 to 2018.

**Figure 1:**
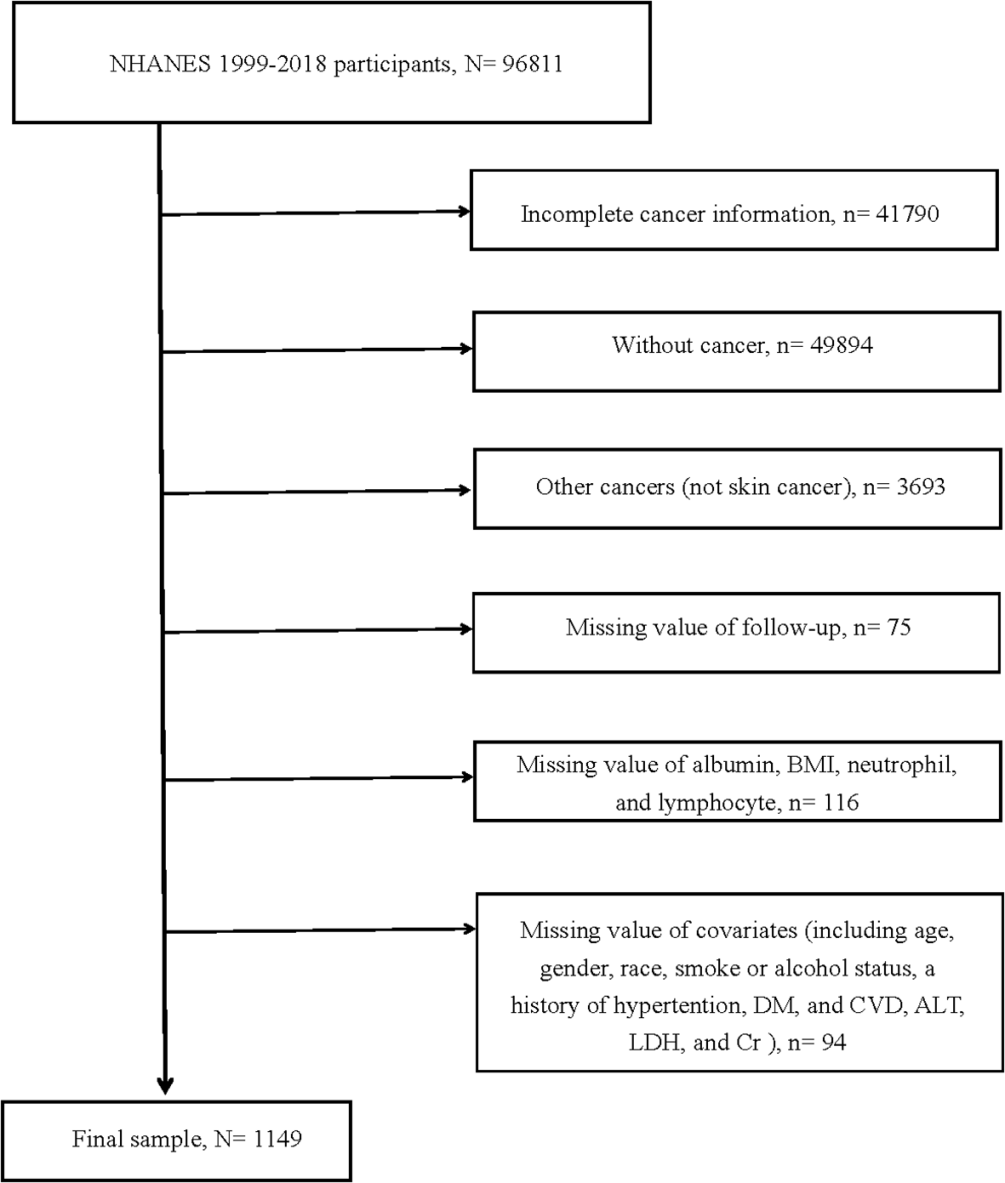
Flow chart of study participants. BMI, body mass index; DM, diabetes mellitus; CVD, cardiovascular disease; ALT, alanine aminotransferase; LDH, lactic dehydrogenase; Cr, creatinine N, The number of patients being included. n, The number of patients being excluded.

Within the 1999–2018 NHANES dataset, our scrutiny was confined to 96,811 participants. From this pool, exclusions were made as follows: 41,790 individuals with incomplete cancer information, 49,894 without a cancer diagnosis, 3,693 diagnosed with cancers other than melanoma or non-melanoma skin cancers, 75 with missing follow-up data, 116 lacking essential values such as albumin, BMI, neutrophils, and lymphocytes. Furthermore, an additional 94 were excluded due to missing covariate data. Consequently, the study cohort was consolidated to encompass 1,149 participants (**Figure 1**).

### Calculation of ALI

The ALI was calculated using the formula: BMI (kg/m^2) × serum albumin (g/dL)/NLR. Patients were stratified into quartiles based on their ALI scores, forming four distinct groups: Q1 group (ALI ≤ 37.87), Q2 group (ALI > 37.87 and ≤ 52.84), Q3 group (ALI > 52.84 and ≤ 73.20), and Q4 group (ALI > 73.20).

### Primary outcome

The primary outcomes of interest were all-cause mortality and cancer mortality. The cause of death was determined using the International Classification of Diseases, 10th Edition (ICD-10) codes. All-cause and cancer mortalities were identified using ICD-10 codes ranging from I00 to I078. For participants included in the NHANES cohort from 1999 to 2018, mortality follow-up data was available up to December 31, 2019.

### Definitions of variables of interest

Demographic variables such as age, gender, and race were self-reported by participants. Laboratory measurements, including alanine aminotransferase (ALT), lactic dehydrogenase (LDH), creatinine (Cr), albumin, neutrophil, and lymphocyte counts, were obtained using standardized automated hematological analysis equipment. The National Center for Health Statistics provided detailed methodologies for these measurements on its website. The BMI was determined by the standard calculation of weight (kg)/[height (m)]^2. Smoking status was categorized based on participant responses; they were identified as never smokers, former smokers, or current smokers according to their smoking history and current smoking habits. Alcohol consumption was assessed and participants were grouped as non-drinkers, former drinkers, mild drinkers, moderate drinkers, or heavy drinkers based on their self-reported alcohol intake. The criteria for hypertension included either taking anti-hypertensive medication or having a mean systolic blood pressure (SBP) ≥ 140 mmHg or a mean diastolic blood pressure (DBP) ≥ 90 mmHg at the time of measurement or a self-reported diagnosis. Diabetes mellitus was defined by the use of hypoglycemic agents or insulin, hemoglobin A1c levels ≥ 6.5%, fasting glucose ≥ 7.0 mmol/L, a random blood glucose ≥ 11.1 mmol/L, a 2-hour oral glucose tolerance test ≥ 11.1 mmol/L, or a self-reported diagnosis. Prediabetes was characterized by fasting blood glucose levels between 6.0-7.0 mmol/L or 2-hour postprandial glucose levels between 7.8-11.1 mmol/L. A history of cardiovascular disease (CVD) was determined by self-reported history of conditions such as congestive heart failure, heart attack, coronary heart disease, angina, or stroke.

### Statistical analyses

We used the NHANES-recommended weights to calculate the appropriate weights for specific groups. Continuous variables were presented as mean ± standard deviation, and for those not following a normal distribution, we represented them by the median (25th percentile, 75th percentile). Categorical variables were reported as counts (percentages). To compared baseline characteristics among the four groups, we applied variance analysis (ANOVA) for continuous variables and the χ2 test for categorical variables.

In analyzing the association between ALI and skin cancer all-cause mortality and cancer mortality, our analysis included Kaplan-Meier and Cox regression analyses, utilizing the NHANES-recommended weights. Model 1 adjusted for demographic factors: age (years), gender (male or female), and race (White, Black, Mexican American, or other). Enhancing Model 1, Model 2 incorporated adjustmented for smoke status (never, former, or current), alcohol consumption (never, former, mild, moderate, or heavy), and disease status (presence or absence, including history of hypertension, diabetes mellitus (DM), and CVD). Further refining our analysis, Model 3 added ALT (U/L), LDH (mmol/L), and Cr (umol/L) to the adjustments made in Model 2. To identify potential non-linear relationships between ALI and all-cause mortality and cancer mortality, we employed restricted cubic splines (RCS). An inflection point was determined from the RCS analysis, and its impact was assessed using segmented Cox analysis. To quantify the ALI’s impact, we divided the ALI levels of each participant by 10, assessing the effect of every 10-unit change in ALI on all-cause mortality and cancer mortality in skin cancer patients. Subsequent COX regression analysis was performed on the variables required for ALI calculation. We also undertooked a stratified analysis to explore the ALI and skin cancer all-cause and cancer mortality relationship across different subgroups, including age, gender, smoke status, hypertension, DM, ALT, and Cr, enhancing the robustness of our findings.

All analyses were conducted using R software (version 4.3.1), with a two-sided P-value of <0.05 designated as the threshold for statistical significance in all analyses.

## Results

### Patient characteristics

Among all 1149 participants who met the study criteria, the average age was 63.13 (62.14, 64.13). The proportion of males was higher (n = 653, 56.83%), with the majority being White (n = 1075, 93.56%). Based on ALI quartiles, patients were divided into four groups: Q1 (n = 288), Q2 (n = 286), Q3 (n = 287), and Q4 (n = 288). The ALI median values for Q4 (105.64), Q3 (45.11), and Q2 (61.61) were higher than for Q1 (29.39).

Participants in the higher ALI groups were younger (Q1: 68.04 vs. Q2: 63.80 vs. Q3: 61.89 vs. Q4: 59.93) and had higher BMI (Q1: 25.64 vs. Q2: 27.13 vs. Q3: 28.78 vs. Q4: 30.88). In the higher ALI groups, participants had lower neutrophil levels (Q1: 5.34 vs. Q2: 4.49 vs. Q3: 4.00 vs. Q4: 3.40) but higher lymphocyte levels (Q1: 1.44 vs. Q2: 1.77 vs. Q3: 1.99 vs. Q4: 2.91). Statistical differences in albumin, ALT, and Cr were also observed across the groups. However, other indicators, including the number of gender, race, LDH, smoking status, alcohol consumption, disease status, and the proportion of skin cancer among the groups, showed no statistically significant difference. More data on the baseline characteristics of the study population can be found in **Table 1**.

**Table 1:** Baseline demographic and medical characteristics of patients with skin cancer in the NHANES 1999–2018 cohort.

Characteristics	ALI					
	Total	Quantile 1 29.74 [4.14,37.87]	Quantile 2 44.73 (37.87,52.84]	Quantile 3 60.98 (52.84,73.20]	Quantile 4 89.14 (73.20,977.87]	*P*
Participants, n	1149	288	286	287	288	
ALI, mean	62.93 (58.87,67.00)	29.39 (28.56, 30.21)	45.11 (44.50, 45.72)	61.61 (60.79, 62.44)	105.64 (94.80,116.49)	< 0.0001
Age, year	63.13 (62.14,64.13)	68.04 (66.16,69.92)	63.80 (61.81,65.79)	61.89 (60.16,63.62)	59.93 (58.20,61.66)	< 0.0001
Gender, n (%)						0.57
Female	496(43.17)	104(44.26)	126(48.56)	120(43.48)	146(49.43)	
Male	653(56.83)	184(55.74)	160(51.44)	167(56.52)	142(50.57)	
Race, n (%)						0.77
White	1075(93.56)	278(97.46)	271(97.69)	265(97.25)	261(96.41)	
Black	15(1.31)	2(0.32)	3(0.39)	3(0.29)	7(0.92)	
Mexican American	25(2.18)	2(0.17)	7(0.59)	8(0.76)	8(0.42)	
Other	34(2.96)	6(2.05)	5(1.33)	11(1.69)	12(2.25)	
BMI, Kg/m2	28.29 (27.87,28.71)	25.64 (24.88,26.40)	27.13 (26.43,27.84)	28.78 (28.01,29.55)	30.88 (30.05,31.72)	< 0.0001
Albumin, g/dL	4.26 (4.24,4.28)	4.18 (4.13,4.23)	4.26 (4.22,4.30)	4.30 (4.27,4.34)	4.30 (4.26,4.33)	< 0.001
Neutrophil, K/uL	4.24 (4.13,4.35)	5.34 (5.03,5.65)	4.49 (4.31,4.67)	4.00 (3.84,4.15)	3.40 (3.25,3.55)	< 0.0001
Lymphocyte, K/uL	2.07 (1.86,2.29)	1.44 (1.36,1.51)	1.77 (1.71,1.83)	1.99 (1.92,2.05)	2.91 (2.20,3.61)	< 0.0001
ALT, U/L	24.36 (23.19,25.53)	22.01 (19.15,24.87)	22.62 (21.10,24.15)	26.55 (23.81,29.29)	25.63 (24.07,27.19)	0.03
LDH, mmol/L	136.94 (134.74,139.13)	142.13 (137.54,146.73)	135.30 (131.33,139.27)	134.71 (131.77,137.66)	136.44 (132.50,140.38)	0.05
Cr, umol/L	83.44 (81.50,85.38)	91.14 (84.48,97.80)	81.81 (78.26,85.36)	82.41 (80.15,84.67)	79.88 (77.13,82.64)	0.03
Smoke status, n (%)						0.86
Never	508(44.21)	132(48.93)	116(43.49)	123(46.10)	137(47.59)	
Former	508(44.21)	126(36.65)	133(43.55)	128(40.53)	121(40.99)	
Now	133(11.58)	30(14.42)	37(12.96)	36(13.37)	30(11.42)	
Alcohol, n (%)						0.11
Never	129(11.23)	43(11.96)	25(7.26)	29(7.55)	32(9.68)	
Former	264(22.98)	76(20.61)	68(19.82)	63(16.95)	57(14.72)	
Mild	532(46.3)	134(50.33)	137(49.75)	128(47.32)	133(46.98)	
Moderate	139(12.1)	17(6.71)	32(13.39)	44(19.56)	46(19.20)	
Heavy	85(7.4)	18(10.40)	24(9.78)	23(8.63)	20(9.43)	
Hypertension, n (%)						0.1
No	435(37.86)	83(34.61)	118(47.10)	113(41.35)	121(45.45)	
Yes	714(62.14)	205(65.39)	168(52.90)	174(58.65)	167(54.55)	
DM, n (%)						0.1
No	763(66.41)	190(71.56)	207(77.41)	185(66.85)	181(68.56)	
preDM	119(10.36)	34(9.28)	19(6.01)	29(9.23)	37(12.93)	
DM	267(23.24)	64(19.16)	60(16.58)	73(23.91)	70(18.51)	
CVD, n (%)						0.1
No	875(76.15)	199(77.60)	217(81.25)	227(86.10)	232(83.53)	
Yes	274(23.85)	89(22.40)	69(18.75)	60(13.90)	56(16.47)	
Skin cancer, n (%)						0.68
Melanoma	234(20.37)	49(18.03)	64(24.15)	61(19.80)	60(19.83)	
Non-melanoma	615(53.52)	148(54.25)	153(54.70)	159(57.20)	155(56.41)	
Skin (unknown)	300(26.11)	91(27.71)	69(21.15)	67(23.01)	73(23.76)	

ALI, advanced lung cancer inflammation index; BMI, body mass index; ALT, alanine aminotransferase; LDH, lactic dehydrogenase; Cr, creatinine; DM, diabetes mellitus; CVD, cardiovascular disease.

Values are weighted mean (IQR) for continuous variables or numbers (weighted %) for categorical variables. Wilcoxon rank-sum test was used for continuous variables, and chi-squared test with Rao & Scott’s second-order correction was used for categorical variables.

### ALI and skin cancer mortality

**Figure 2:**
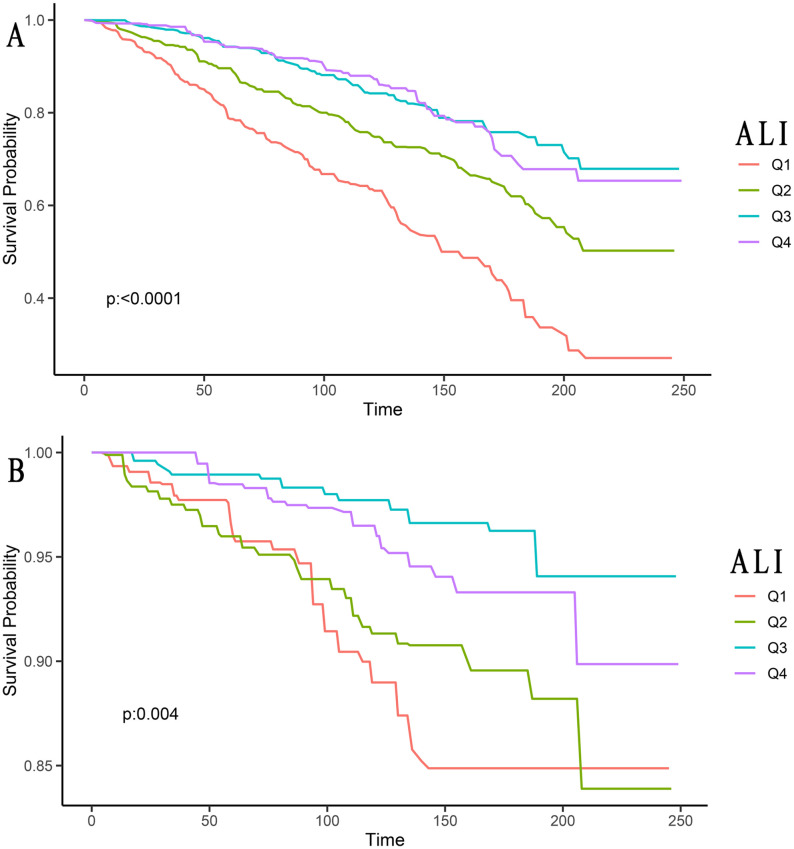
Kaplan-Meier survival curves of ALI impact on long-term all-cause mortality **(A)**, and cancer mortality **(B)** in patients with skin cancer (weighted). ALI, advanced lung cancer inflammation index; Q1, Quantile 1; Q2, Quantile 2; Q3, Quantile 3; Q4, Quantile 4.

Among the 1,149 skin cancer patients included, there were 234 (20.37%) with melanoma, 615 (53.52%) with non-melanoma, and 300 (26.11%) with Skin (unknown). These were divided into Q1, Q2, Q3, and Q4 groups using quartile methods for Kaplan-Meier survival analysis curves. From the graph, we could see that ALI was correlated with both all-cause mortality and cancer mortality in skin cancer patients (all *P*-values <0.05, **Figure 2**).

The univariate Cox proportional hazard results (**Table 2**) showed that, compared to the Q1 group, the all-cause mortality risk in skin cancer patients in the Q2 group (HR: 0.54, 95% CI: 0.38–0.77), Q3 group (HR: 0.30, 95% CI: 0.21–0.43), and Q4 group (HR: 0.31, 95% CI: 0.22–0.44) decreased by 46%, 70%, and 69%, respectively. This difference was statistically significant (*P* for trend <0.001).

**Table 2:** Relationships of ALI with all-cause and cancer mortality in patients with skin cancer from the NHANES 1999–2018 cohort.

ALI	All -cause mortality			
	Crude	Model 1	Model 2	Model 3
Skin cancer*	HR, 95%CI	HR, 95%CI	HR, 95%CI	HR, 95%CI
Quantile 1	ref	ref	ref	ref
Quantile 2	0.54(0.38,0.77)	0.80(0.59,1.08)	0.75(0.56,1.01)	0.77(0.57,1.05)
Quantile 3	0.30(0.21,0.43)	0.51(0.37,0.70)	0.48(0.35,0.66)	0.50(0.36,0.69)
Quantile 4	0.31(0.22,0.44)	0.60(0.42,0.84)	0.53(0.37,0.74)	0.54(0.38,0.77)
***P* for trend**	<0.001	<0.001	<0.001	<0.001
	Cancer mortality			
	Crude	Model 1	Model 2	Model 3
Skin cancer*	HR, 95%CI	HR, 95%CI	HR, 95%CI	HR, 95%CI
Quantile 1	ref	ref	ref	ref
Quantile 2	0.84(0.46,1.54)	1.16(0.65,2.08)	1.18(0.65, 2.16)	1.20(0.66, 2.18)
Quantile 3	0.28(0.12,0.63)	0.40(0.17,0.94)	0.38(0.16, 0.89)	0.38(0.16, 0.89)
Quantile 4	0.42(0.20,0.91)	0.67(0.31,1.47)	0.64(0.30, 1.38)	0.64(0.30, 1.35)
***P* for trend**	0.005	0.090	0.060	0.049

ALI, advanced lung cancer inflammation index; ref, reference; HR, hazard ratios; CI, confidence interval; CVD, cardiovascular disease; ALT, alanine aminotransferase; LDH, lactic dehydrogenase; Cr, creatinine.

Values are n or weighted HR (95% CI). Model 1: adjusted for age (years), gender (male or female), and race or ethnicity (White, Black, Mexican American, or other). Model 2: model 1+ adjusted for smoke status (never, former, or now), alcohol (never, former, mild, moderate, or heavy), and disease status (yes or no, including a history of hypertension, diabetes mellitus, and CVD). Model 3: model 2+ adjusted for ALT (U/L), LDH(mmol/L), and Cr(umol/L).

* Skin cancer, including melanoma, non-melanoma, and skin (unknown type).

After adjusting for potential confounding factors such as age, gender, race, smoking status, alcohol consumption, hypertension, DM, CVD, ALT, LDH, and Cr, compared to the Q1 group, the all-cause mortality risk in skin cancer patients in the Q2 group (HR: 0.77, 95% CI: 0.57–1.05), Q3 group (HR: 0.50, 95% CI: 0.36–0.69), and Q4 group (HR: 0.54, 95% CI: 0.38–0.77) still showed varying degrees of reduction. This difference was statistically significant (*P* for trend <0.001).

Similarly, both the unadjusted and adjusted Cox proportional hazard results showed that, compared to the Q1 group, the cancer mortality risk in skin cancer patients in the Q2, Q3, and Q4 groups all decreased to varying degrees. This difference was statistically significant (*P* for trend = 0.005 and *P* for trend = 0.049).

### The detection of the non-linear relationship

**Figure 3:**
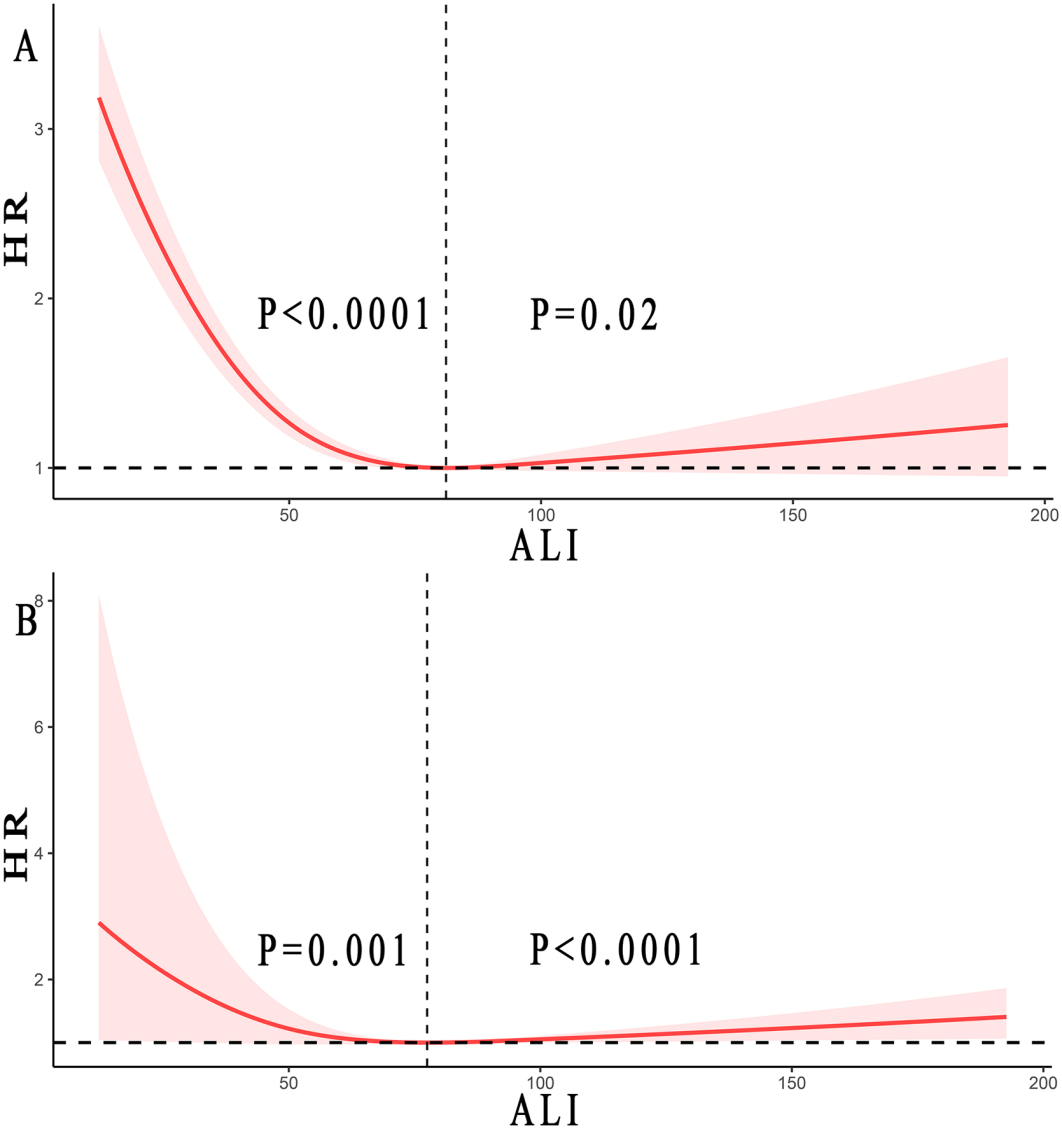
Relationship between ALI and all-cause mortality **(A)** and cancer mortality **(B)** in patients with skin cancer. Adjusted for age, gender, race, smoke status, alcohol, a history of hypertension, diabetes mellitus, and CVD, ALT, LDH (mmol/L), and Cr (umol/L). The solid and red shadow represent the estimated values and their 95% CIs, respectively. ALI, advanced lung cancer inflammation index; CVD, cardiovascular disease; ALT, alanine aminotransferase; LDH, lactic dehydrogenase; Cr, creatinine. Regarding all-cause mortality, when ALI was below 81.13, the P <0.0001, while when ALI exceeded 81.13, the P =0.02. For cancer mortality, when ALI was less than 77.50, the P =0.001, whereas when ALI was greater than 77.50, the P <0.0001.

Through restricted cubic splines analysis combined with the Cox proportional hazards model, we found a reverse J-shaped non-linear relationship between ALI and all-cause mortality and cancer mortality in skin cancer, as shown in **Figure 3**. Our results indicated that the inflection point for all-cause mortality in skin cancer was 81.13, and for cancer mortality, it was 77.50.

When the ALI was above the inflection point, an increase of 10U in ALI resulted in a 2% and 6% increased in the multivariate-adjusted HR for all-cause mortality and cancer mortality, respectively (HR 1.02, 95% CI: from 1.00 to 1.04 and HR 1.06, 95% CI: from 1.05 to 1.07). On the other hand, when the ALI was below the inflection point, an increase of 10U in ALI resulted in a 20% and 21% decrease in the multivariate-adjusted HR for all-cause mortality and cancer mortality, respectively (HR 0.80, 95% CI: from 0.74 to 0.86 and HR 0.79, 95% CI: from 0.66 to 0.95), as shown in **Table 3**.

**Table 3:** Threshold effect analysis of ALI on all-cause, and cancer mortality in patients with skin cancer.

	All -cause mortality	
	Per 10U increment	*P*
Skin cancer*		
<81.13	0.80 (0.74,0.86)	<0.0001
>81.13	1.02 (1.00, 1.04)	0.02
	Cancer mortality	
	Per 10U increment	*P*
Skin cancer*		
<77.50	0.79 (0.66,0.95)	0.001
>77.50	1.06 (1.05,1.07)	<0.0001

ALI, advanced lung cancer inflammation index; CVD, cardiovascular disease; ALT, alanine aminotransferase; LDH, lactic dehydrogenase; Cr, creatinine.

Values are n or weighted HR (95% CI). Model is adjusted for age (years), gender (male or female), race or ethnicity (White, Black, Mexican American, or other), smoke status (never, former, or now), alcohol (never, former, mild, moderate, or heavy), disease status (yes or no, including a history of hypertension, diabetes mellitus, and CVD), ALT (U/L), LDH(mmol/L), and Cr(umol/L).

*Skin cancer, including melanoma, non-melanoma, and skin (unknown type).

### The stratified and sensitivity analyses

When participants were stratified by age (*P* for interaction = 0.12), gender (*P* for interaction = 0.22), smoking status (*P* for interaction = 0.63), hypertension (*P* for interaction = 0.43), DM (*P* for interaction = 0.67), ALT (*P* for interaction = 0.67), and Cr (*P* for interaction = 0.15), the association between ALI and all-cause mortality did not change. Sensitivity analysis showed that among individuals with Cr levels ≥106 umol/L, those with ALI greater than 81.37 had a 68% reduced risk of all-cause mortality in skin cancer patients (HR 0.32, 95% CI: from 0.14 to 0.87, *P* for trend = 0.01). No other indicators had significant associations. Similarly, when participants were stratified by age (*P* for interaction = 0.27), gender (*P* for interaction = 0.71), smoking status (*P* for interaction = 0.39), hypertension (*P* for interaction = 0.65), DM (*P* for interaction = 0.63), and Cr (*P* for interaction = 0.59), the association between ALI and cancer mortality did not change. Sensitivity analysis showed that the results for ALI and cancer mortality in skin cancer were consistent with the main effects (Additional file 1).

## Discussion

To our knowledge, this was the first large-scale study of ALI and skin tumors to investigate the relationship between ALI and long-term health outcomes in patients with skin cancer. We observed an inverse J-shaped non-linear relationship between ALI and both all-cause and cancer mortality. Specifically, when the ALI was above the inflection point, an increase of 10U in ALI resulted in a 2% and 6% increased in the multivariate-adjusted HR for all-cause mortality and cancer mortality, respectively. Conversely, when the ALI was below the inflection point, an increase of 10U in ALI led to a 20% and 21% decreased in the multivariate-adjusted HR for all-cause mortality and tumor mortality, respectively.

Skin cancer is one of the most common malignant tumors, primarily caused by prolonged exposure to ultraviolet radiation. It is also influenced by the patient’s susceptibility related to their skin type (17). Basal cell carcinoma is a low-grade malignant epithelial tumor that originates in the basal cells of the epidermis or the outer root sheath of hair follicles. It typically progresses slowly and has a lower incidence of distant metastasis compared to other malignant skin tumors. However, its incidence has been steadily increasing in recent years. Squamous cell carcinoma is another type of malignant skin tumor that arises from epidermal keratinocytes. Although its incidence is lower than that of basal cell carcinoma, it has a higher rate of distant metastasis and mortality. In contrast, melanoma, while having the lowest incidence among skin cancers, carries a very high risk of metastasis and significantly higher mortality compared to the previous two types. Recent research indicated that early detection of skin cancer was crucial, as it led to the highest relative survival rates. Therefore, the early diagnosis and detection of skin cancer hold significant research significance and practical value (18).

Inflammation and malnutrition associated with cancer played crucial roles in tumor progression, and the prognosis of cancer largely depended on the baseline inflammation and nutritional status of the patient. Recent years have seen a growing body of evidence highlighting the connection between inflammation and the onset and progression of cancer (19, 20); Systemic inflammation could manifest in changes in peripheral blood leukocytes, which could be quantified using the NLR (21). Recent studies have suggested that melanoma patients with a high NLR tend to have a poorer prognosis (22, 23).

Malnutrition and cachexia were significant concerns in cancer patients, as they involved various mechanisms related to tumor development, the host’s response to the tumor, and anti-tumor treatments (20). Several methods existed to assess the nutritional status of cancer patients, along with numerous indicators for evaluating nutritional status, such as hematocrit, hemoglobin, albumin, transferrin, heme, serum creatinine, urine creatinine, and BMI, among others (24). In clinical practice, the most commonly used nutritional indicators are albumin and BMI. The impact of albumin on the prognosis of skin cancer was evident. Cancer patients often experience cachexia due to inadequate nutrient intake and tumor-related consumption. Serum albumin could promptly reflect changes in the patient’s nutritional status, and hypoalbuminemia often occurred as cancer progresses (25). Many scholars have found that BMI was closely related to the prognosis of skin cancer (26, 27). However, research results on the relationship between BMI and skin cancer were inconsistent (28–30).

ALI incorporated multiple values, including BMI, serum albumin, absolute neutrophil count in peripheral blood, and absolute lymphocyte count. These values effectively reflected the patient’s nutritional, immunological, and overall inflammatory status. Initially used to assess systemic inflammation in patients diagnosed with metastatic non-small cell lung cancer (14). ALI was now increasingly employed in the study of various clinical tumors (31–33).

Our research results demonstrated a correlation between ALI levels and the prognosis of skin cancer. This finding aligned with the previous discoveries by Xi Cheng and colleagues (34), indicating that ALI could function as an independent predictive biomarker for the prognosis of metastatic melanoma with immunotherapy. What distinguishes our study was that, for the first time on a large sample scale, we explored the relationship between ALI and skin cancer, revealing an inverse J-shaped non-linear relationship between ALI and both all-cause and cancer mortality. Below the inflection point, all-cause and cancer mortality decreased as ALI increased, while above the inflection point, both all-cause and cancer mortality increased with higher ALI levels. The complex non-linear relationship between ALI and skin cancer might be attributed to the composition of the ALI index.

However, this study has some limitations. Firstly, it was an observational study, and despite the large sample size, it could not definitively establish a causal relationship between ALI and mortality in skin cancer patients. The causality between ALI and mortality should be confirmed through future interventional studies with large samples. Secondly, despited our efforts to eliminate biases, there might still be unknown confounding factors.

In summary, as a comprehensive assessment of patients’ nutritional and inflammatory status, ALI revealed an inverse J-shaped non-linear relationship with all-cause and cancer mortality. This suggested that maintaining the appropriate level of ALI might have a certain effect on improving the prognosis of patients, thereby providing some new ideas for clinical research.

## Data Availability

The original contributions presented in the study are included in the article/**Supplementary Material**. Further inquiries can be directed to the corresponding authors.
