# Supplementary material for: Advanced lung cancer inflammation index is associated with prognosis in skin cancer patients: a retrospective cohort study
Source: Front Oncol. 2024 Oct 11;14:1365702. doi: 10.3389/fonc.2024.1365702 (PMC11502321; doi:10.3389/fonc.2024.1365702)

**Supplementary Online Content**

**Supplementary Methods**

**Supplementary Table S1.** Stratified analyses of the relationships of ALI with all-cause mortality in patients with skin cancer from the NHANES 1999–2018 cohort.

**Supplementary Table S2.** Stratified analyses of the relationships of ALI with cancer mortality in patients with skin cancer from the NHANES 1999–2018 cohort.

**Supplementary Table S3.** Stratified analyses of threshold effect about the relationships of ALI with all-cause mortality in patients with skin cancer from the NHANES 1999–2018 cohort.

**Supplementary Table S4.** Stratified analyses of threshold effect about the relationships of ALI with cancer mortality in patients with skin cancer from the NHANES 1999–2018 cohort.

**Supplementary Figure S1.** The forest plot of ALI and skin cancer mortality by selected subgroups.

**Supplementary Table S1. Stratified analyses of the relationships of ALI with all-cause mortality in patients with skin cancer from the NHANES 1999–2018 cohort**

| **Characteristics** | **ALI** | | | | | |
| --- | --- | --- | --- | --- | --- | --- |
|  | **Quantile 1**  29.74  [4.14,37.87] | **Quantile 2**  44.73  (37.87,52.84] | **Quantile 3**  60.98  (52.84,73.20] | **Quantile 4**  89.14  (73.20,977.87] | ***P* for trend** | ***P* for interaction** |
| Participants, n | 288 | 286 | 287 | 288 |  |  |
| Age |  |  |  |  |  | 0.12 |
| <60 | ref | 0.31(0.07,1.39) | 0.21(0.03,1.54) | 0.67(0.15,3.03) | 0.75 |  |
| ≥60 | ref | 0.78(0.58,1.04) | 0.42(0.30,0.59) | 0.40(0.27,0.59) | <0.0001 |  |
| Gender |  |  |  |  |  | 0.22 |
| Male | ref | 0.62(0.41,0.92) | 0.46(0.310.70) | 0.39(0.24,0.64) | <0.0001 |  |
| Female | ref | 0.91(0.55,1.52) | 0.30(0.16,0.57) | 0.51(0.31,0.83) | <0.001 |  |
| Smoke status |  |  |  |  |  | 0.63 |
| Never | ref | 0.71(0.44,1.14) | 0.36(0.21,0.61) | 0.33(0.19,0.56) | <0.0001 |  |
| Former | ref | 0.69(0.45,1.07) | 0.38(0.24,0.59) | 0.37(0.22,0.63) | <0.0001 |  |
| Now | ref | 1.00(0.29,3.49) | 0.71(0.15,3.30) | 1.12(0.25,4.96) | 0.99 |  |
| Hypertension |  |  |  |  |  | 0.43 |
| No | ref | 0.61(0.35,1.07) | 0.42(0.19,0.90) | 0.57(0.28,1.16) | 0.15 |  |
| Yes | ref | 0.74(0.53,1.04) | 0.39(0.26,0.60) | 0.36(0.23,0.55) | <0.0001 |  |
| DM |  |  |  |  |  | 0.67 |
| No | ref | 0.75(0.50,1.12) | 0.48(0.29,0.81) | 0.44(0.28,0.68) | <0.0001 |  |
| PreDM | ref | 0.76(0.27,2.18) | 0.51(0.19,1.35) | 0.34(0.14,0.87) | 0.03 |  |
| DM | ref | 0.55(0.29,1.08) | 0.23(0.12,0.43) | 0.39(0.20,0.77) | 0.001 |  |
| ALT |  |  |  |  |  | 0.29 |
| <40 | ref | 0.73(0.53,0.99) | 0.41(0.28,0.60) | 0.42(0.29,0.62) | <0.0001 |  |
| ≥40 | ref | 0.47(0.09,2.33) | 0.07(0.01,0.59) | 0.41(0.12,1.46) | 0.02 |  |
| Cr |  |  |  |  |  | 0.15 |
| <106 | ref | 0.66(0.46,0.96) | 0.32(0.21,0.47) | 0.40(0.27,0.59) | <0.0001 |  |
| ≥106 | ref | 0.83(0.53,1.29) | 0.71(0.36,1.39) | 0.35(0.15,0.78) | 0.01 |  |

**Supplementary Table S2. Stratified analyses of the relationships of ALI with cancer mortality in patients with skin cancer from the NHANES 1999–2018.**

| **Characteristics** | **ALI** | | | | | |
| --- | --- | --- | --- | --- | --- | --- |
|  | **Quantile 1**  29.74  [4.14,37.87] | **Quantile 2**  44.73  (37.87,52.84] | **Quantile 3**  60.98  (52.84,73.20] | **Quantile 4**  89.14  (73.20,977.87] | ***P* for trend** | ***P* for interaction** |
| Participants, n | 288 | 286 | 287 | 288 |  |  |
| Age |  |  |  |  |  | 0.27 |
| <60 | ref | 3.81( 0.50,28.84) | 0.00( 0.00,0.00) | 0.35( 0.05,2.65) | 0.05 |  |
| ≥60 | ref | 1.11(0.58,2.12) | 0.43(0.18,1.04) | 0.59(0.26,1.32) | 0.06 |  |
| Gender |  |  |  |  |  | 0.71 |
| Male | ref | 1.03(0.48, 2.23) | 0.43(0.17, 1.12) | 0.57(0.24, 1.35) | 0.08 |  |
| Female | ref | 0.95(0.32, 2.82) | 0.17(0.04, 0.70) | 0.42(0.13, 1.29) | 0.03 |  |
| Smoke status |  |  |  |  |  | 0.39 |
| Never | ref | 0.90(0.26, 3.13) | 1.05(0.22, 5.02) | 0.60(0.13, 2.77) | 0.63 |  |
| Former | ref | 0.94(0.42, 2.12) | 0.27(0.09, 0.77) | 0.47(0.18, 1.22) | 0.03 |  |
| Now | ref | 8.48(0.99, 72.95) | 0.13(0.00, 9.20) | 5.06(0.56,45.42) | 0.55 |  |
| Hypertension |  |  |  |  |  | 0.65 |
| No | ref | 0.92(0.25, 3.31) | 0.20(0.04, 1.10) | 0.71(0.22, 2.34) | 0.4 |  |
| Yes | ref | 1.14(0.56, 2.31) | 0.40(0.16, 1.02) | 0.51(0.20, 1.27) | 0.03 |  |
| DM |  |  |  |  |  | 0.63 |
| No | ref | 1.13(0.53, 2.43) | 0.36(0.11, 1.15) | 0.36(0.13, 0.99) | 0.01 |  |
| PreDM | ref | 2.67(0.43,16.56) | 0.28(0.02, 3.26) | 1.13(0.19, 6.66) | 0.68 |  |
| DM | ref | 0.58(0.11, 2.95) | 0.35(0.08, 1.46) | 0.75(0.18, 3.22) | 0.77 |  |
| Cr |  |  |  |  |  | 0.59 |
| <106 | ref | 1.16(0.54, 2.47) | 0.33(0.13, 0.89) | 0.52(0.22, 1.20) | 0.02 |  |
| ≥106 | ref | 0.75(0.23, 2.48) | 0.46(0.07, 3.10) | 0.87(0.20,3.77) | 0.71 |  |

**Supplementary Table S3. Stratified analyses of threshold effect about the relationships of ALI with all-cause mortality in patients with skin cancer from the NHANES 1999–2018 cohort**

| **Characteristics** | **ALI** | | | |
| --- | --- | --- | --- | --- |
|  | <81.37 | >81.37 | ***P* for trend** | ***P* for interaction** |
| Age |  |  |  | 0.08 |
| <60 | ref | 2.42(0.73,8.02) | 0.15 |  |
| ≥60 | ref | 0.74(0.50,1.11) | 0.15 |  |
| Gender |  |  |  | 0.53 |
| Male | ref | 0.90(0.54,1.49) | 0.67 |  |
| Female | ref | 0.88(0.53,1.46) | 0.62 |  |
| Smoke status |  |  |  | 0.12 |
| Never | ref | 0.56(0.34,0.91) | 0.02 |  |
| Former | ref | 0.82(0.48,1.37) | 0.44 |  |
| Now | ref | 2.43(1.18,5.03) | 0.02 |  |
| Hypertension |  |  |  | 0.47 |
| No | ref | 1.20(0.66,2.21) | 0.55 |  |
| Yes | ref | 0.73(0.46,1.16) | 0.19 |  |
| DM |  |  |  | 0.86 |
| No | ref | 0.80(0.49,1.29) | 0.36 |  |
| PreDM | ref | 0.76(0.34,1.72) | 0.52 |  |
| DM | ref | 1.04(0.57,1.91) | 0.89 |  |
| ALT |  |  |  |  |
| <40 | ref | 0.87(0.58,1.31) | 0.51 | 0.77 |
| ≥40 | ref | 1.16(0.47,2.88) | 0.75 |  |
| Cr |  |  |  | 0.03 |
| <106 | ref | 0.99(0.67,1.45) | 0.95 |  |
| ≥106 | ref | 0.32(0.14,0.75) | 0.01 |  |

**Supplementary Table S4. Stratified analyses of threshold effect about the relationships of ALI with cancer mortality in patients with skin cancer from the NHANES 1999–2018 cohort**

| **Characteristics** | **ALI** | | | |
| --- | --- | --- | --- | --- |
|  | <77.50 | >77.50 | ***P* for trend** | ***P* for interaction** |
| Age |  |  |  | 0.68 |
| <60 | ref | 0.82(0.17,3.92) | 0.81 |  |
| ≥60 | ref | 0.88(0.43,1.80) | 0.73 |  |
| Gender |  |  |  | 0.94 |
| Male | ref | 0.87(0.39,1.94) | 0.73 |  |
| Female | ref | 1.02(0.39,2.69) | 0.96 |  |
| Smoke status |  |  |  | 0.41 |
| Never | ref | 0.73(0.17,3.10) | 0.67 |  |
| Former | ref | 0.82(0.36,1.90) | 0.65 |  |
| Now | ref | 2.08(0.53,8.11) | 0.29 |  |
| Hypertension |  |  |  | 0.76 |
| No | ref | 1.23(0.47,3.22) | 0.67 |  |
| Yes | ref | 0.83(0.37,1.86) | 0.66 |  |
| DM |  |  |  | 0.33 |
| No | ref | 0.56(0.22,1.39) | 0.21 |  |
| PreDM | ref | 1.62(0.42,6.25) | 0.48 |  |
| DM | ref | 1.30(0.33,5.22) | 0.71 |  |
| Cr |  |  |  | 0.35 |
| <106 | ref | 0.82(0.41,1.62) | 0.56 |  |
| ≥106 | ref | 1.64(0.46,5.81) | 0.44 |  |

**Supplementary Figure S1.** The forest plot of ALI and skin cancer mortality by selected subgroups.


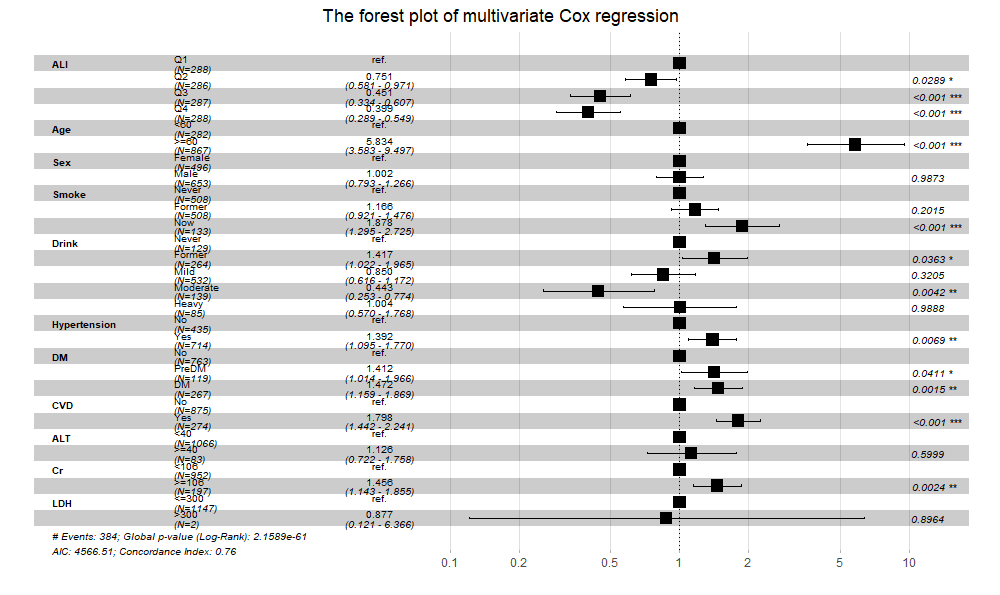

Supplement: Supplementary file 1 [file DataSheet1.doc]
